# Editorial: Shaping of Human Immune System and Metabolic Processes by Viruses and Microorganisms

**DOI:** 10.3389/fmicb.2019.00816

**Published:** 2019-04-17

**Authors:** Marina I. Arleevskaya, Rustam Aminov, Wesley H. Brooks, Gayane Manukyan, Yves Renaudineau

**Affiliations:** ^1^Central Research Laboratory, Kazan State Medical Academy, Kazan, Russia; ^2^School of Medicine and Dentistry, University of Aberdeen, Aberdeen, United Kingdom; ^3^Department of Chemistry, University of South Florida, Tampa, FL, United States; ^4^Group of Molecular and Cellular Immunology, Institute of Molecular Biology, National Academy of Sciences, Yerevan, Armenia; ^5^Laboratory of Immunology and Immunotherapy, INSERM U1227, University of Brest, Brest, France

**Keywords:** microbiota, immune system, autoimmunity, cancer, allergy, viruses

## Introduction

**Figure 1:**
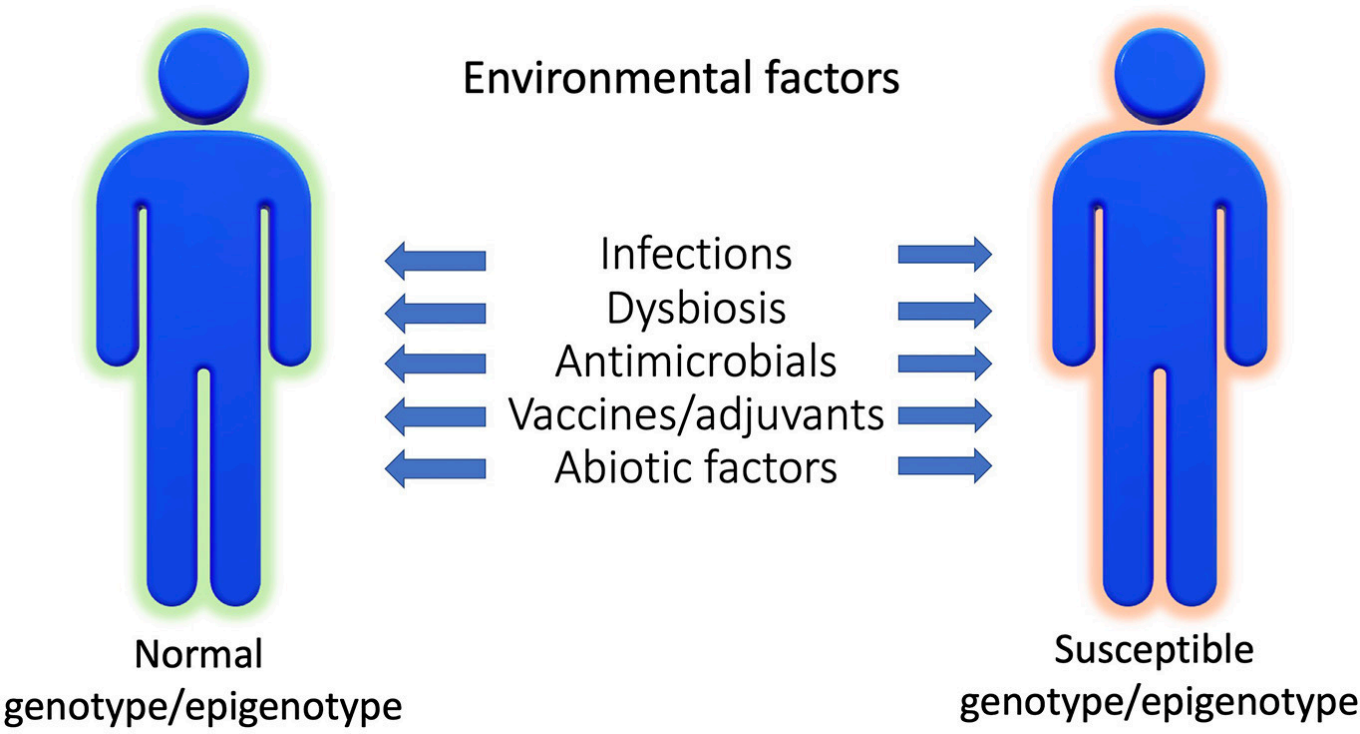
Disease phenotype as a result of interaction between genetic/epigenetic and environmental factors.

**Figure 2:**
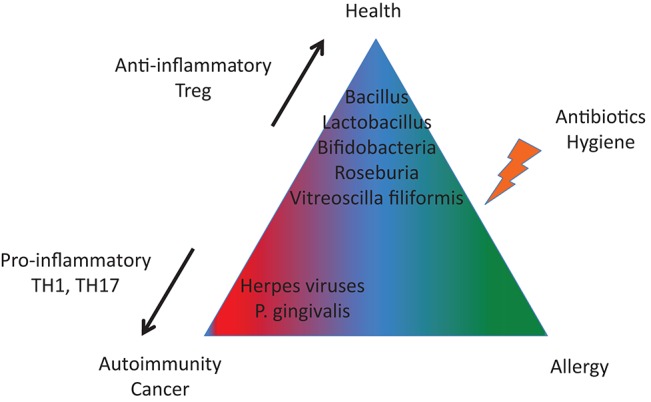
A tight equilibrium between infectious agents is important to protect against autoimmunity and cancer (imbalanced in terms of pro-inflammatory and anti-inflammatory bacterial species) and against allergies (altered microbiota).

We are not alone in our bodies since we share them with a huge number of microorganisms. Such interactions represent a continuum, extending from mutualistic relationships, to commensal interactions and, at the end of the spectrum, development of human diseases (Lerner et al.). While substantial progress has been made in our understanding of the pathophysiology of infectious diseases, more subtle interactions exist with the microbiome and those interactions could promote or protect against the development of human disease according to the genetic/epigenetic susceptibility of the individual (Figure 1). By interfering with our bodies, modulating our immune system, controlling our metabolism, even our mood, microorganisms and viruses can actively contribute to the development of diseases that are major causes of mortality, and in particular by promoting cancers and autoimmune processes (Figure 2). At the same time, there is also the flip side of the coin as the indigenous microflora inhibits colonization by exogenous pathogens through both bacterial antagonism and host immune system stimulation. While and according to the hygiene hypothesis, a defective indigenous microflora and/or a lack of infectious agent exposure in childhood increase susceptibility to allergic diseases by suppressing the natural development of the immune system.

We offer this Research Topic involving 63 articles and 500 authors to discuss recent advances regarding:

1. An overview of the human microbiota and its capacity to interact with the human immune system and metabolic processes,
2. New developments in understanding the immune system's strategies to respond to infections and escape strategies used by pathogens to counteract such responses,
3. The link between the microbiota and pathology in terms of autoimmunity, allergy, cancers and other diseases.

## Relationships Between Microbiota, Viruses and the Host

### Gut Microbiota

The gut microbiota results first from a tight equilibrium between the need for beneficial microbiota to ferment and digest incoming dietary material and the need to exclude competing pathogens. In addition, the gut microbiota depends on the individual's characteristics such as birth delivery mode, immune system activation state, host genetics and metabolism, and history of diseases; and to the influence of environmental factors, namely standards of hygiene, familial relationships, medication exposure including antibiotics, hospitalization after birth, lifestyle, cultural aspects, and contamination of food and water by fecal microbes; that all can alter transmission mechanisms.

What is the connection between the human microbiome and the soil microbial community? This obvious question is reviewed by Tasnim et al. Data analyses retrieved from published reports show that overlap between gut and soil and microbes is limited based on the observation that two phyla, Bacteriodetes and Firmicutes, dominate human fecal samples, whereas dominant phyla in soil samples are Proteobacteria and Verrucomicrobia. However, such a statement needs to be taken with caution since most soil and gut surveys were performed in North American cohorts, and it was not possible to determine whether this can be transposed to other geographical locations.

What is the impact of the human birth delivery mode on the gut microbiota? To answer this question, Stewart and coauthors have studied longitudinal development of the gut microbiome of preterm infants (24–31 weeks gestation) during the first 100 days of life following either cesarean or vaginal discharge (Stewart et al.). The main conclusion from their study is that there is no significant association of birth mode with the longitudinal alpha- or beta- diversity or composition of the microbiome. One possible explanation came from the fact that all infants were exposed to antibiotics at postpartum.

Toscano and colleagues have reviewed the importance of the human breast milk microbiota in the formation of the newborn's first gut microbiota during lactation which is a determining factor in the maturation of the immune system of newborns (Toscano et al.). Human breast milk microbiota is comprised of more than 200 different bacterial species and one key question is related to their origin as some microorganisms (*Streptococcus* spp. and *Staphylococcus* spp.) belong to the maternal skin or infant's oral cavity, suggesting a milk flow back into mammary ducts during lactation. Human breast milk contains also a great number of intestinal bacteria, which may spread from the maternal intestinal environment by a particular mechanism involving dendritic cells and neutrophils (Rodríguez and Wilson, 2014).

### Other Microbiota

Microbiota analysis is not restricted to the gut as Baker and coauthors summarized with regards to the current status of uterine microbiota (Baker et al.). The notion that a healthy uterine cavity is sterile is currently being revised since the uterine microbiota contains 100–10,000 times less bacteria than the vaginal microbiome. The most abundant uterine bacteria found consistently belong to the following phyla: Firmicutes, Bacteriodetes, Proteobacteria, and Actinobacteria. Within the Firmicutes, the genus Lactobacillus is the prominent one. Another emerging question regarding uterine microbiota in women is related to the transmission routes: (i) hematogenous spread through either an oral or gut route; (ii) ascension through the cervix; (iii) retrograde spread through fallopian tubes; and (iv) assisted reproductive technology-related procedures or insertion/removal of intra-uterine devices. Such a list needs to be extended since Altamae suggests to add the seminal contribution in the uterine microbiota (Altmae).

What about human skin microbiota? Park and Lee discussed the protective role of the commensal skin and orogenital microbiota in protecting the host from chronic immune-mediated inflammatory disease (Park and Lee). Indeed, commensal microbial species are effective for controlling (i) release of antimicrobial peptides by skin cells, (ii) the pro-inflammatory microbial sensor NOD2 (nucleotide-binding oligomerization domain-containing protein 2) pathway; and (iii) components of the complement system. In particular, *Staphylococcus epidermidis* as well as *Corynebacterium pseudodiphtheriticum, Propionibacterium acnes*, and *Staphylococcus aureus* impact in a “compartmentalized” manner IL-17A production and skin-resident Th17 cells (Pascal et al., 2018). Another example is *Vitreoscilla filiformis*, a Gram-negative bacterium, which induces dendritic cells to prime naive T cells to type 1 Treg cells after cutaneous exposure (Volz et al., 2014).

Regarding human oral microbiota, Vieira and colleagues discussed the impact of estrogen deficiency associated with menopause (Vieira et al.). Such an assertion is linked, on one hand, with the description of the estrogen receptor-beta in the oral mucosa and salivary glands (Välimaa et al., 2004), and, on the other hand, with the observation that menopause was associated in women with age-related hormonal changes in the exfoliated normal buccal mucosa. Specific bacterial species, such as *Porphyromonas gingivalis* and *Tannerella forsythensis*, were found to be important in the etiology of periodontitis in postmenopausal women. The presence or absence of estrogen is also suspected to alter the gut microbiota equilibrium and to promote intestinal permeability. Another unsuspected link between the gut microbiome and menopausal health is related to bone since, for instance, *Lactobacillus reuteri* treatment prevents post-antibiotic and post-menopausal bone loss by reducing the expression of two inflammatory cytokines (TNF-α and IL-1β), increasing osteoclastogenesis, and preventing barrier disruption (Britton et al., 2014; Schepper et al., 2019).

An investigation into the interplay between the oral cavity microbiome in humans with the host immune system has also been reported (Park and Lee; Vieira et al.). In particular it was demonstrated that *P. gingivalis*, a member of the phylum Bacteroidetes, manipulate the local host responses by preventing phagocytosis and rerouting the anti-microbial Toll-like receptor (TLR) pathway after acting on both TLR2 and C5a receptor (C5aR) (Maekawa et al., 2014). *P. gingivalis* inhibits the secretion of IL-8, lowering the number of neutrophils recruited to the site of inflammation. *P. gingivalis* is also known to maintain a hyper-inflammatory state by enhancing M1 macrophages and a TH1/TH17 immune response, which has been attributed in part to the ability to secrete the *P.gingivalis*-derived peptidyl arginine deiminase (PPAD) that opens up the possibility of converting arginine residues into citrulline residues. A loss of tolerance against citrullinated proteins can lead to the development of rheumatoid arthritis as reviewed by Sakkas and colleagues (Arleevskaya et al., 2016; Sakkas et al.).

As this editorial and the related special issue did not cover the entire microbiota spectrum, readers are invited to complete their overview with recent publications presenting the growing interest in the virome and parasitome in human conditions (Marzano et al., 2017; Mitchell and Glanville, 2018).

### Interplay Between Microbiota and the Immune System

Based on the observation that germ-free mice present an altered immunity with increased susceptibility to immunological diseases and metabolic alterations, Spiljar and coauthors reviewed the interconnection between gut microbiota, the immune system and systemic energy homeostasis (Spiljar et al.). The absence of gut microbiota leads (i) to the formation of isolated secondary lymphoid follicles and smaller Payer's patches; (ii) to decreased counts of immune cells; and (iii) to a reduced local production of immunoglobulin. Such an observation is linked to functional alterations in immune and intestinal epithelial cells, and in particular a down-regulation of the anti-microbial TLR pathway. At the same time germ-free mice show improved glucose and insulin tolerance, accompanied by reduced adiposity. The metabolic effects and the browning phenotype are mediated by the innate immune system and the shift from the pro-inflammatory M1 to the anti-inflammatory M2 macrophage polarization.

Another way to test the relationship of the microbiome on the immune system is to induce microbiota depletion by antibiotics and then explore the impact on the immune system after bacterial recolonization with *E. coli, L. johnsonii* or with fecal microbiota transplantation (FMT) as performed in mice by Ekmekciu et al. First, immune cell populations were decreased after antibiotic treatment but were completely or at least partially restored upon FMT or by recolonization with the respective bacterial species. In particular (i) *L. johnsonii* recolonization results in the highest CD4+ and CD8+ cell numbers in the small intestine and spleen; (ii) FMT most effectively increases the frequencies of Treg cells, dendritic cells and restores intestinal memory/effector T cell populations; (iii) *E. coli* recolonization increases the production of TH1/TH17 cytokines, particularly in the small intestine; and conversely (iv) *L. johnsonii* recolonization maintains colonic IL-10 production. In summary, FMT appears in this mouse model to be efficient in the restoration of antibiotic-induced collateral damage to the immune system. Similarly, the use of the environmental toxicant 2,3,7,8-tetrachlorodibenzo-p-dioxin (TCDD), an aryl hydrocarbon receptor (AhR), induces a shift in the gut microbiota by interacting with AhR ligand bacteria such as segmented filamentous bacteria, an immune activator, as presented by Stedtfeld et al.

Patterson and coauthors, using murine and *in vitro* models, have investigated the mechanisms of cross-talk between the host immune system and the anti-inflammatory human gut symbiont *Roseburia hominis* (Patterson et al.). In the bacterium, host gut colonization upregulated *R. hominis* genes involved in conjugation/mobilization, metabolism, motility, and chemotaxis. In the host cells, *R. hominis* colonization upregulated genes related to antimicrobial peptides, gut barrier function, TLR5-flagellin signaling, and Treg population expansion.

The *Lactobacillus* group is classically regarded as “beneficial” in humans, however this group is in a competitive relationship with other bacteria as reported by Rodríguez-Carrio and coauthors who have studied the impact of the main gut microbial populations (*Akkermansia, Bacteroides sp, Bifidobacterium, Clostridium cluster XIVa, Lactobacillus sp, and Faecalibacterium*) on fecal short-chain fatty acid (SCFA), serum free fatty acid (FFA) levels, and immune pro-inflammatory mediators (IFNγ, IL-6, and MCP-1) in healthy human adults (Rodriguez-Carrio et al.). From this study an opposing behavioral pattern between *Akkermansia* (negative relation) and *Lactobacillus* (positive relation) was demonstrated by the authors regarding their relation with FFA, SCFA, and pro-inflammatory IL-6 levels. The modulation of the immune response by lipid metabolites including SCFA is developed by Shibata et al., and a comprehensive analysis of SCFA in the gut microbiota based on the diet is presented by Soverini et al. Within SCFA, some have particular effects on the immune system such as propionic acid that modulates mitochondrial dysfunction with an impact on leukocyte antigen expression and immunoglobulin production as studied *in silico* by Frye et al.

Ilinskaya and coauthor's present arguments to consider that secreted compounds from two probiotics of the *Bacilli* class, genera *Bacillus* and *Lactobacillus*, can affect both the human microbiome composition and the immune system (Ilinskaya et al.). Indeed, these two probiotics are able to secrete compounds, referred to as the bacillary secretome, and which are effective in suppressing pathogenic bacteria and favor beneficial ones via competition for nutrients, especially for shared limited resource like iron, competitive attachment to the epithelium, formation of substrates for growth, production of waste products and antimicrobial compounds, and strengthening of the barrier function of the epithelium. Regarding their interaction with the immune system, the bacillary secretome controls key signaling pathways such as TLR, NF-κB, and MAPK pathways and, in turn, the emergence of pro-inflammatory cytokines is reduced for the benefit of anti-inflammatory cytokines.

Mu and coauthors reviewed the probiotic properties of *Lactobacillus reuteri* in humans (Mu et al.). First, *L. reuteri* produces antimicrobial molecules that are able to inhibit the colonization of pathogenic microbes and to remodel the commensal microbiota composition in the host. Second, *L. reuteri* can benefit the host immune system. For instance, some *L. reuteri* strains reduce the production of pro-inflammatory cytokines while promoting Treg cell development and function. Third, bearing the ability to strengthen the intestinal barrier, the colonization of *L. reuteri* decreases the microbial translocation from the gut lumen to the tissues.

Ruiz and coauthors discussed the immunomodulatory properties of Bifidobacteria and the mechanisms and molecular players underlying these processes (Ruiz et al.). Currently it is accepted that there is a critical role for bifidobacteria in the maturation of the host innate and acquired immune system from gestation to childhood. Contrarily, other bacteria such as Mycoplasma infections elicit inflammatory as well as immuno-modulatory effects through surface lipoproteins as reviewed by Christodoulides et al.

## Immune System

### Immune Response and Altered Immune Response During Infections

Ghazarian and coauthors comprehensively characterized a population of mucosal-associated invariant T (MAIT) cells in humans, which are unconventional CD3+ CD161^high^ T lymphocytes that recognize a bacterial vitamin B2 (riboflavin) precursor presented by the MHC-I related protein, MR1 (Ghazarian et al.; Shibata et al.). MAIT cells represent an abundant proportion of resident T cells in tissues (10–40%) and up to 10% of the circulating T cell pool. Upon TCR-dependent recognition of microbial antigens, MAIT cells secrete inflammatory cytokines (IFNγ, TNF-α, IL-17, and sometimes IL-22) and perforin to induce cellular cytotoxicity against bacterially infected cells. Human MAIT cells can also be activated *in vitro* in a TCR-MR1 independent fashion during acute or chronic viral infections in response to cytokines such as IL-12, IL-18, IL-15, and/or interferon α/β.

Khaiboullina et al. have studied cytokine levels in patient sera samples collected during acute and late phases of hemorrhagic fever with renal syndrome (HFRS) and hantavirus pulmonary syndrome (HPS), two primary forms of Hantavirus infection (Khaiboullina et al.). The authors report a cytokine storm in both diseases and with particularly a robust TH1 and NK cell response in HPS.

The innate and adaptive immune responses to Epstein-Barr virus (EBV) are not fully understood and, due to the complexity of the mammalian immune system, Sherri and coauthors propose to use the insects *Drosophila melanogaster* in order to better characterize innate immune pathways that are activated in response to EBV DNA (Sherri et al.). Back to human, Lam and colleagues traced the dynamics of antigen-specific poly-functional CD4 and CD8 T cell responses against lytic and latent antigens of EBV in children diagnosed to have primary symptomatic infectious mononucleosis and asymptomatic EBV infection (Lam et al.). Elevated viral loads, which declined steadily during a 12-month period from the time of diagnosis, is reported whilst a decrease in the magnitude of the CD8 cytotoxic T cell response with the greatest response being toward immuno-dominant epitopes in both lytic and latent proteins, is correlated to a steady decline in viral loads.

Starting from the observation that idiopathic infertile women present both a modified percentage of endometrial (e)NK cells and infection with the human herpes virus (HHV)-6A, Casseli and colleagues have explored the capacity of HHV-6 virus to induce cytotoxic eNK cells as such activation may have implications for embryo implantation and infertility (Caselli et al.). This hypothesis is supported by the observation that eNK cells infected with HHV-6 have an increased expression of chemokines (CCR2, CXCR3, and CX3CR1) and those endometrial epithelial cells up-regulated the corresponding ligands (MCP1, CXCL10, and CCL26). Another consequence of infection with HHV-6A is related to its impact on the complement pathway. Indeed, HHV-6A infects target cells by docking to the complement regulator CD46, which results in CD46 downregulation, a lack that can lead to hyperactivation of the complement pathway. This mechanism described in astrocytes may explain the connection reported between HHV-6A infection and age-related macular degeneration (AMD) (Fierz).

### Escape Strategies: Innate Response

Chen and coauthors discuss how the influenza A virus (IAV, Orthomyxoviridae family) has developed multiple strategies to escape from host immune surveillance for successful replication (Chen et al.). First, an exceptional variability of the most abundant surface glycoprotein of the virus, hemagglutinin (HA), delays the production of neutralizing antibodies by B cells. Second, neuraminidase (NA), the second most abundant surface glycoprotein, reduces infected cell recognition by NK cells through its capacity to cleave sialic acid (SA) moieties. Third, the nonstructural protein-1 (NS1) acts as an important interferon antagonist protein. Fourth, the viral M2 protein is able to block host autophagy by interfering with the TLR-interferon pathway. So, the host immune response to IAV infection comprises multiple intricate processes that coordinate together to play significant roles in the protection of the virus.

Rodriguez and coauthors have reviewed the impact of chronic viral infection on the control of the human T-cell dependent humoral immune response in secondary lymphoid organs (Rodriguez et al.). Such an impact varies according to the cellular partner implicated: B cells, follicular helper T cells (Tfh), and stromal cells with two main subsets: fibroblast reticular cells (FRC) involved in B and T cell recruitment and follicular dendritic cells (FDC) involved in the formation and maintenance of the germinal center. The first example is EBV that infects mature B cells and mimics BCR and CD40 signals in the absence of antigen by producing the latent membrane proteins LMP1/2. As a consequence EBV drives B cell proliferation in a germinal center independent way that leads to memory B cells infected by EBV that are not recognized by the immune system. The second example is HIV that infects CD4 T cells and proliferates predominantly in Tfh that become defective in driving an effective anti-HIV humoral response. The third example is related to Ebola, Lassa and Marburg Viruses plus the lymphocytic choriomeningitis virus that destroy FRC leading to lymph node disorganization and a defective humoral and cellular immune response. The fourth example is arboviruses that infect and kill FDC thus affecting the capacity of the germinal center to produce protective antibodies and inducing a transitory cellular immuno-depression.

### Escape Strategies: miRNAs and lncRNAs

Human herpes xeno-miRNAs regulate host cell proliferation, differentiation, apoptosis, and the cell cycle to establish latent viral infection or, contrarily, to produce viral reactivation progeny as discussed by Kim et al. When secreted, xeno-miRNAs result in additional stimulation to the activation of the pro-inflammatory intracellular signaling and to the repression of various immune responses that can shift host cells to malignant transformation.

Rizzo and coauthors in an original research article have explored the impact of HHV-6 infection on the host miRNAs in NK cells (Rizzo et al.). To this end, a human NK cell line (NK-92) was infected with HHV-6A/B *in vitro*. HHV-6A/B induced significant modification in miRNA expression which are known for their role in inflammation, and NK cell development, maturation and effector functions (miR-146, miR-155, miR-181, miR-223). Similarly Kaul and coauthors have performed a comprehensive analysis of differentially expressed host peripheral blood miRNAs in the early stage of uncomplicated EBV infection in patients with acute infectious mononucleosis (Kaul et al.). They identified 215 differentially regulated miRNAs at the most acute stage of infection. Interferon signaling, T and B cell signaling and antigen presentation were the top pathways influenced by the miRNAs associated with this acute EBV infection. Altogether, this supports the idea that herpes viruses, HHV-6 and EBV, have developed an escape strategy by controlling host miRNA expression.

During the interferon response, several long non-coding RNAs (lncRNAs) are induced to control positively or negatively the expression of interferon-stimulated genes (ISGs) in order to stop the interferon response and to return to a normal state. This negative regulatory mechanism can be subverted for the virus's benefit by viruses such as hepatitis C virus (HCV) as reviewed by Valadkhan and Fortes.

### Other Escape Strategies

Zhang and co-authors reviewed another mechanism of interference used by members of the Flaviviridae family, HCV and dengue virus (DENV) (Zhang et al.). Since viruses lack the appropriate machinery to conduct their own lipid synthesis, they have evolved a means to interfere with the cellular homeostasis of lipid droplets in the endoplasmic reticulum. The authors also discuss the possibility of targeting the host lipid droplet metabolism as antiviral strategies. Besides allowing *Helicobacter pylori* survival within the gastric mucosa, *H. pylori* urease has an important pro-inflammatory effect on both neutrophils *via* ROS production and platelet aggregation as proposed by Scoppel-Guerra and colleagues (Scopel-Guerra et al.), as well as on angiogenesis as supported by Olivera-Severo et al.

Malisheni and colleagues have conducted meta-analysis to clarify clinical efficacy, safety, and immunogenicity of a Live Attenuated Tetravalent Dengue vaccine (CYD-TDV) in children (Malisheni et al.). The analysis demonstrated that CYD-TDV is rather effective against viral serotypes DENV4, DENV3, and DENV1, and sufficiently less effective against DENV2 serotype. The low vaccine efficacy could be due to the fact that the circulating DENV2 virus acquires mutations in the E protein hence becoming different from the one included in the CYD-TDV vaccine.

## Link With Diseases

### Autoimmunity

Disbalanced gut microbiota is suspected of influencing a large panel of autoimmune diseases as reviewed by Opazo and Picchianti-Diamant (Picchianti-Diamanti et al.; Opazo et al.). In the case of inflammatory bowel disease (IBD), one way to improve the disease is to reduce gut permeability by restoring commensal gut microbiota. To this end, Li and coauthors have used the colitis-mice model to test the suitability of 10 strain bacterial consortium transplantation (BCT) (Li et al.). It was demonstrated that BCT resulted in the reduction of gut permeability and improvement of intestinal dysbiosis as well as in the elevation of IL-17A producing γδT cells in colonic lamina propria of mice. In addition, the expansion of γδT17 cells was related to *Bifidobacterium sp* and *Bacillus sp* in a TLR2 dependent pathway. Another way to control gut dysbiosis is to use a high-fat, adequate-protein, low-carbohydrate diet, namely a ketogenic diet, as reported in patients with multiple sclerosis by Swidsinski et al.

Arleevskaya and coauthors have reported an increased peak of infectious episodes at early stages of rheumatoid arthritis (eRA) development and in their first-degree relatives (FDR) when compared to healthy women without RA in their family history (Arleevskaya et al.; Arleevskaya et al., 2018). A history of herpes simplex virus (HSV) episodes and reactivation is associated with inflammation and disease activity (Arleevskaya et al.; Larionova et al., 2019), while an elevated incidence of anti-CCP2 autoantibody characterized eRA patients with a history of viral upper respiratory tract infection symptoms. Regarding the innate immune system, granulocyte reactive oxygen species (ROS) activity is altered in eRA patients, associated with viral symptoms including HSV exacerbation/recurrence, and such defects are positively correlated with disease activity. In addition to the well establish association between periodontitis due to *P. gingivallis* and RA, a meta-analysis conducted by Rutter-Locher and collaborators supports also an association with systemic lupus erythematosus, another systemic autoimmune disease (Rutter-Locher et al.).

### Allergy and Cancers

As described for microbiota, commensal viruses control innate and adaptive immune defenses and an imbalance of the virome is thought to trigger the development of allergic diseases, like asthma which is an allergic airway inflammation. As a consequence, restoring the virome represents an attractive strategy to control allergies as proposed by Freer and colleagues based on the use of *Anelloviridae*, a recently discovered virus family and major component of the respiratory virome acquired early in life (Freer et al.). Another way is to control asthma by modulating the gut microbiota and this was effective when using *Bifidobacterium longumin* in ovariectomized allergic mice with albumin as reported by Mendes et al., This prophylactic effect of *B. longumin* involves fecal acetate production and, in turn, Treg induction. Type I hypersensitivity reactions can be seen in bronchial asthma and the diagnosis of allergic bronchopulmonary aspergillosis can be improved based on the use of anti-*Aspergillus fumigatus* (Af) IgE, IgG, and IgG4 biomarkers as proposed by Vitte et al.

The development of cancer can be promoted by infections through different mechanisms. First, an altered oral and gut microbiota can directly affect the inflammatory and carcinogenic response of the host as presented by Klimesova et al. Second and as presented by Blumenthal et al., a defective immune response to cancer can be promoted by the synergic effect of infectious agents like the human immunodeficiency virus (HIV) and an altered metabolic process such as type 2 diabetes. Hyperglycemia and type 2 diabetes contribution to lymphoma reactivation is also commented on by Angius et al. Third, an enhanced humoral response to infectious agents based on the characteristics of the monoclonal immunoglobulin associated with multiple myeloma development as proposed by Bosseboeuf et al. Monoclonal IgG purified from multiple myeloma patients are able to target pathogens known to cause latent infection; they are desialylated to better exert their anti-infectious effect; and they promote an inflammatory cytokine response including cytokines important for the anti-microbial response.

### Other Diseases

Dysbalanced human gut microbiota is suspected of promoting inflammatory diseases and one proposed mechanisms is that the microbiota itself or its metabolism are able to influence gut permeability and, in turn, to promote endotoxin translocation into the blood circulation. The first example is provided by Saltzman and colleagues who discussed the composition of the gut microbiome and its biological consequences in regard to liver homeostasis and liver inflammatory diseases (Saltzman et al.), the impact of long-term intake of fructose is also discussed by Lambertz et al. The second example is proposed by Sanchez-Alcoholado and colleagues as the production of trimethylamine N-oxide (TMAO) from the gut microbiota promotes the development of cardiovascular diseases and impacts the production of the anti-inflammatory cytokine IL-10 and Treg development (Sanchez-Alcoholado et al.). The third example by Zhao and colleagues is related to the observation that a major source of pro-inflammatory signals in Alzheimer's disease (AD), at the brain neocortex and hippocampus, arises from pro-inflammatory neurotoxic bacterial lipopolysaccharide (LPS) secreted from Gram negative bacilli of the human gastrointestinal (GI) tract (Zhao et al.).

Host and microbiome share similar or identical nutritional substrates and generate common metabolic products, thus a metabolic cross-talk exists with profound implications regarding (i) the pathogenesis of an infection as presented by Ren et al.; (ii) the immune system as discussed previously; and (iii) the risk to develop a metabolic syndrome according to Pindjakova and colleagues when using a non-inflammatory diet-mimicking healthy obesity or a pro-inflammatory and atherogenic paigen diet to fed mice (Pindjakova et al.).

## New Models and Hypothesis

In order to better understand the cross-talk between microbiota and the immune system in complex systems, such as the gut or skin, new models are proposed based on the use of genetic and epigenetic *in silico* approaches as performed by Li et al., the use of bacteriophages to selectively target a species as claimed by Kłopot et al., or *in vivo* models based on the use of microinjections in the ear pinna dermis in transgenic mice with fluorescent immune cells as elegantly developed by Forestier et al.

Finally, in a review article, Lerner and colleagues address an important issue regarding fundamental genetic processes in the commensal gut microbiota, horizontal gene transfer (HGT), and how they may affect human health (Lerner et al.) or contribute to the emergence of hyper-virulent and drug resistant pathogens as reported by Khaertynov et al. Another important question is related to the putative impact of viruses on the nucleolus as such disruption can lead to generation of autoantigens as discussed by Brooks et al. (2010), Brooks and Renaudineau (2015), and Brooks. Finally, Douville and Nath in their review discussed a particular aspect of human immunodeficiency virus (HIV)-associated neurological conditions, such as HIV encephalitis and HIV-associated neurocognitive disorders, mediated by neuronal human endogenous retrovirus-K (HERV-K) expression (Douville and Nath). This reinforces the contribution of HERV elements in human diseases (Renaudineau et al., 2005; Fali et al., 2014; Le Dantec et al., 2015).

## Conclusions

In conclusion, articles in this Research Topic made a very significant contribution to our understanding of the role played by environmental factors, dysbiotic conditions, and infections in triggering diseases. Since this is a rapidly expanding area of research, many other factors contributing to the onset of these diseases are not covered here. We are confident, however, that further studies will expand the list as well as bring a better understanding of mechanisms involved in the onset of autoimmune and autoinflammatory diseases.

## Author Contributions

All authors listed have made a substantial, direct and intellectual contribution to the work, and approved it for publication.

### Conflict of Interest Statement

The authors declare that the research was conducted in the absence of any commercial or financial relationships that could be construed as a potential conflict of interest.
